# Comprehensive evaluation of transgenic rice lines expressing CSFV E2 protein in genetic stability, environmental safety and field adaptation

**DOI:** 10.3389/fpls.2025.1650765

**Published:** 2025-12-02

**Authors:** Hongyan Chu, Lei Zhang, Zhen Hao, Yupeng Hua, Wenjing Hu, Ruijing Liu, Yanyue Lou, Wenming Gao, Shuangli Bian, Fei Li, Lichuang Han, Wenbo Cheng, Jiangnan Zhang, Yi Zhu, Shiyuan Pan, Shaokang Kou, Hengyao Chen, Erqin Zhang, Xuannian Wang, Gaiping Zhang

**Affiliations:** 1 International Joint Research Center of National Animal Immunology, College of Veterinary Medicine, Henan Agriculture University, Zhengzhou, China; 2 Longhu Laboratory of Advanced Immunology, Zhengzhou, China; 3 School of Advanced Agricultural Sciences, Peking University, Beijing, China; 4 Institute for Animal Health, Henan Academy of Agricultural Sciences, Zhengzhou, China

**Keywords:** rice endosperm expression, genetic stability, agronomic traits, biosafety, plant-derived vaccine, swine fever virus

## Abstract

Classical swine fever virus (CSFV) is a virulent pathogen that affects the global swine industry. Classical swine fever caused by CSFV is highly lethal and transmissible. Current attenuated vaccines present challenges, such as biosafety concerns and limitations in differential diagnosis. In previous study, we successfully constructed transgenic rice strains, E2-1 and E2-2, expressing the CSFV E2 antigen using a high-efficiency bioreactor system based on rice endosperm. This platform provides a safe and cost-effective approach for large-scale oral subunit vaccine production due to the protein-storage properties of rice endosperm and the absence of contamination risks from animal pathogens. In this study, systematic tracking of T1–T3 plant generations confirmed stable integration and continuous expression of the E2 gene, as verified by PCR and Western blot, with antigen titers in seeds remaining stable up to 2^7. Agronomic traits analysis revealed that the transgenic lines and the low-gluten rice variety showed significant improvements in plant height, grain number per spike, grain chalkiness, and other key indexes, thereby maintaining strong cultivation adaptability. Environmental safety tests demonstrated that the transgenic lines E2-2were more suitable for E2 vaccine production and posed no risk of genetic drift to surrounding weeds. This study is the first to report assessments of genetic stability, agronomic performance, and environmental safety of the CSFV E2 antigen expressed in multiple rice generations, which lays a key technical foundation for the commercial development of plant-derived swine fever vaccine.

## 1 Introduction

Viruses of the genus Distemper virus of the Flaviviridae family are a serious threat to animal health worldwide and cause significant economic losses. Classical swine fever virus (CSFV) was the first reported pathogen caused by distemper viruses, which are single-stranded positive-stranded RNA capsid viruses. Outbreaks of classical swine fever (CSF) are characterised by high mortality rates, severe immunosuppression, and rapid transmission (Paton and Greiser-Wilke, 2003; van Oirschot, 2003; Luo et al., 2014; Blome et al., 2017a, Blome et al., 2017b; Fahnøe et al., 2019; Ganges et al., 2020), posing a significant threat to the swine industry (Khairullah et al., 2024). Currently, widely used CSF vaccines, such as primary cell-based and passaged cell-based, rely on mammalian or insect cell culture systems (Lin et al., 2009, Lin et al., 2012). These systems are expensive, show considerable batch-to-batch variability in antigenic content, and present a risk of contamination with exogenous viruses (e.g., bovine viral contaminants). In addition, traditional vaccines require cold-chain storage and transportation, limiting their accessibility in resource-limited areas. The co- occurrence of African swine fever (ASF) and CSF further highlights the urgent need to develop novel vaccine platforms (Wei et al., 2021).

In recent years, plant-based bioreactor systems have emerged as promising platforms for the production of recombinant protein drugs and vaccines due to their low-cost production, high biosafety, and minimal cold-chain storage and transport requirements (Giddings et al., 2000; Lomonossoff and D’Aoust, 2016; Jung et al., 2022), Among these systems, the rice endosperm expression platform is particularly noteworthy. Its tissue-specific promoters (e.g., *GluA-2*, *Gt1*) can efficiently drive the accumulation of exogenous proteins in seeds, which naturally possess the ability to stably store proteins at room temperature for extended periods (Shimamoto and Kyozuka, 2002; Rice et al., 2005; Yuki and Kiyono, 2008; Sang et al, 2013; Main et al., 2015; Stander et al., 2022; Zhu et al., 2022; Zahmanova et al., 2023). More importantly, Agrobacterium-mediated genetic transformation allows for the precise integration of exogenous genes into the rice genome, resulting in transgenic lines with stable genetic backgrounds and excellent agronomic traits (Ou et al., 2014). These characteristics make rice endosperm an ideal vehicle for the large-scale production of subunit vaccines for animals, while overcoming the biosafety and process complexity challenges associated with conventional vaccine manufacturing (Fausther-Bovendo and Kobinger, 2021).

Previously, we expressed the swine fever E2 protein in rice endosperm and conducted immunization experiments on pigs. The results showed that a low dose of 284 ng of the E2 vaccine induced neutralising antibody titres 32 times higher than those generated by the live attenuated vaccine. Moreover, the E2-specific antibody response lasted for more than 180 days (Xu et al., 2023). In addition, plant-based vaccine production effectively avoids the risk of exogenous viral contamination associated with traditional virus culture systems (Dalsgaard et al., 1997; Laughlin et al., 2019; Capell et al., 2020; Jung et al., 2022). The commercial application of plant-derived vaccines must be supported by rigorous and systematic scientific evaluation. In this context, the actual value of transgenic crops mainly depends on three core dimensions: genetic stability, agronomic performance, and field biosafety.

This study aims to overcome the technical limitations of traditional vaccines by providing an environmentally friendly and cost-controllable plant-derived vaccine solution for the prevention and control of swine fever. It also establishes a technological paradigm for developing vaccines against livestock and poultry diseases using crop-based bioreactor systems. Furthermore, it promotes cross-disciplinary innovation between plant synthetic biology and the fields of veterinary and animal husbandry sciences.

## 2 Materials and methods

### 2.1 Materials

Tested Plant: transgenic rice lines E2-1 and E2-2; Control variety: low-gluten; Measuring tools: measuring tape (Easy, Cat. No. 713319, 3m); digital vernier caliper (Cat. No. DL91150).

### 2.2 Rice cultivation process

In this rice planting, a uniform field management model (outdoor field) was adopted. The detailed cultivation process of rice is as follows:

(1)Seedling stage: after sunshine seeds will be screened full of rice grain, pharmaceutical (Ethylicin) soaked for about 12 h to the seeds completely absorb water expansion, water control for 1 day and germination.

(2)Management in the shed after planting: weeding (using rice straw to prevent barnyard grass), fertiliser, temperature control, ventilation when growing to ‘two leaves and one heart’ (early side winds, afternoon shutdown), watering changed from 1 day to 2 days, the end of the seedling stage after 30 days.

(3)Transplanting and field management: plough and level the ground after selecting the land, and insert 30 seedlings trays of per mu (Rice row spacing of 30 cm and plant spacing of 16 cm). With 50 cm spacing between all three types of rice strains, namely, low gluten, E2-1 (50 cm spacing between different generations) and E2-2 (50 cm spacing between different generations). Fertiliser is applied 3 days after transplanting according to the seedling condition (less for weak seedlings and more for strong seedlings), and 1 week and 20 days later, 1 time each. During the seedling period, dry the water and then enter the water to promote rooting; after tillering, dry the water to stop tillering and provide nutrients to the effective spike. The whole period according to the growth trend to dry the field, when the soil moisture content of 40% -50% (foot tread on the cracks) when the second into the water, to ensure that the root system permeability.

### 2.3 DNA extraction from rice leaves and spikes

DNA samples were extracted from flowering rice spike tissues of T1-T3 generation transgenic rice lines and Low-glutein plants, which were stored at -80°C. DNA extraction was performed using the FastPure^®^ Plant DNA Isolation Mini Kit (DC104-01, Vazyme), following the manufacturer’s instructions. The extracted DNA was stored at -20°C. DNA from surrounding weeds was extracted using the same procedure. PCR analysis was used to detect the inheritance of the target gene. Primers sequences specific to the E2 gene are listed in **Table 1**. The reaction system for the E2 gene is shown in **Table 2**. The optimal reaction conditions for PCR were as follows: (1) pre-denaturation at 95°C for 5 min; (2) 35 cycles of amplification at 90°C for 30 s, 60°C for 20 s, and 72°C for 30 s; (3) final extension at 72°C for 5 min; (4) storage at 16°C. PCR products were analyzed by agarose gel electrophoresis.

**Table 1:** Name of primer.

Primer	Primer sequence (5′-3′)	Lengths
E2-F	AAGGAGGACTACCGCTACG	958bp
E2-R	GGTACTCGCCCTTGAGC	

**Table 2:** PCR reaction system (50µL).

Reaction system	Volumes (50µL)
2xRapid Taq master mix (P222-01,Vazyme)	25µL
ddH2O	Add to 50µL
E2-F	1µL
E2-R	1µL
cDNA	2µL

### 2.4 Acquisition and qPCR detection of cDNA in rice leaves

T1-T3 generation transgenic and low-gluten plants were selected at the panicle flowering stage. The leaves of rice were transported from Xinjiang to Henan on dry ice and stored at -80°C. Total RNA was extracted using the FastPure^®^ Universal Plant Total RNA Isolation Kit (Cat: RC411-01, Vazyme). cDNA was sythesized via reverse transcription using the HiScript^®^ II Q RT SuperMix for qPCR (+gDNA wiper) (Cat: R223-01, Vazyme), following the manufacturer’s instructions. The cDNA extracted from leaves of T1–T3 generation transgenic rice and low-gluten rice was used as the template, and amplified by qPCR using primer pairs for the target genes and the HygR genes. The primers used were listed in **Table 3**, and the qPCR reaction system is shown in **Table 4**. Relative expression levels of the exogenous and HygR genes in leaves were determined using the 2^-ΔΔCt^ method.

**Table 3:** Name of primer.

Primer	Primer sequence (5′-3′)
E2-F	AAGGAGGACTACCGCTACG
E2-R	GGTGACGCTGGTGAGGA
HygR-F	CTTCTGCGGGCGATTT
HygR-R	ACTGGAGCGAGGCGATG

**Table 4:** qPCR reaction system (20µL).

Reaction system	Volumes (20µL)
2xChamQ Universal SYBR qPCR Master Mix	10µL
ddH_2_O	Add to 20µL
E2-F	0.4µL
E2-R	0.4µL
cDNA	2µL

### 2.5 Western blot

To identify the expression of the E2 protein in mature rice seeds at the protein level, T1-T3 generation transgenic seeds and low-gluten seeds harvested simultaneously and stored under identical conditions were used. Seeds from different lines were ground into powder, then mixed with extraction buffer at a ratio of 1:5 (w/v, g/mL). The mixture was stirred for 1.5 h, followed by centrifugation at 9000 r/min for 30 min at 4°C. The resulting supernatant was obtained for Western blot analysis. Protein markers were purchased from Henan Xianyan Biotech Co., Ltd. (Cat. No. ArP01201) and Vazyme (Lot No. 7E0452L4). Primary antibodies were maintained in the laboratory. The secondary antibody (dilution 1:5,000) was purchased from Proteintech (Cat. No. SA00001-5).

### 2.6 Test strips for detecting antigen content in rice

Antigenic titers were determined in the E2-1 and E2-2 rice lines over three consecutive generations. Immunochromatographic test strips developed in the laboratory were used to detect the E2 antigen in harvested rice seeds. Rice extracts were prepared by mixing the ground seeds with extraction buffer at a mass-to-volume ratio of 1:5 (w/v), followed by stirring at room temperature for 2h. The mixture was then centrifuged at 4°C to collect the supernatant, referred to as the 2^0 dilution. The extracts were subsequently diluted by multiplicative dilution and used in antigen detection assays.

### 2.7 Evaluation of germination and seedling emergence rates

The materials used for evaluating germination and seedling emergence included T1-T3 generation transgenic lines and wild-type seeds harvested simultaneously and stored under identical conditions. For each strain, 100 seeds were placed in 50 mL conical flasks and soaked in water at 25°C in the dark for 36 h. The water was replaced every 12 h to keep cleanliness. After, the seeds were transferred to 9-cm glass Petri dishes lined with moistened sterile filter paper and incubated at 37°C until radicle emergence. Once more than 80% of the seeds showed radicle protrusion through the seed coat, they were transferred to a 25°C incubator and moistened regularly to promote germination. Germination was assessed every 12h and recorded when root buds were visible. When the shoot reached half the grain length, the seeds were transplanted into moist nutrient soil in the culture room. Seedling emergence data were collected and recorded. Throughout the experiment, seeds were kept consistently moist. Germination and seedling emergence rates were calculated as percentages.

### 2.8 Developmental cycle analysis

The developmental cycle analysis was performed using T1-T3 generation transgenic transformants and wild-type control seeds harvested simultaneously and stored under identical conditions. Seeds were sterilized and germinated in medium. After germination, seedlings were transferred to a culture room and then potted in soil. After 20 days, seedlings were moved to outdoor and kept well-watered. The developmental cycle was monitored, tracking both vegetative and reproductive growth stages, with the spikelet stage marking the transition between these phases.

### 2.9 Evaluation of comprehensive agronomic traits

Agronomic traits related to the growth pattern were comprehensively evaluated at the rice maturity stage, defined as the point at which more than 90% of the spike hulls turned yellow and the basal seeds became hard and unbreakable. At this stage, rice spikes from each line were collected into the corresponding numbered seed bags. After sun-drying for 3 days under outdoor conditions, agronomic traits related to spikes and grains were measured.

The recorded agronomic traits related to growth pattern and their specific definitions were as follows: plant height, defined as the distance from the base of the plant to the tip of the second tallest leaf; effective tillers, referring to the number of tillers bearing spikes with more than five mature seeds, counted from the base upward; flag leaf length, measured from the base to the tip of the flag leaf; flag leaf width, indicating the maximum width of the flag leaf; and single-plant weight, which refers to the weight of the entire plant (with roots) after cleaning and blotting dry with a paper towel to remove surface moisture.

The relevant agronomic traits recorded for rice spikes and their specific definitions were as follows: effective spike number, defined as the number of spikes with more than five mature grains per plant; spike length, the distance from the neck node to the tip of an effective spike; effective spike weight, the weight of the effective spike on a single plant; grain number per spike, the total number of grains in an effective spike; fruiting rate, the percentage of filled grains in an effective spike; grain density, the number of grains per centimeter of spike length; and thousand-grain weight, the weight of 1,000 filled grains.

The recorded seed quality traits and their specific definitions were as follows: brown rice percentage, the ratio of brown rice weight (after hull removal) to the total grain weight (before hull removal); grain length, the average length of 10 grains; grain width, the average width of 10 grains; grain thickness, the average thickness of the rice grains; and chalkiness, the proportion of the white, opaque portion in the rice grain, calculated based on the chalkiness rate under fluorescent light and the average area of the chalky portion.

### 2.10 Scanning electron microscopy observation of rice seeds

Rice grains from lines E2-1, E2-2, and Low-gluten were selected. Intact grains were gently fractured using tweezers to expose a flat cross-section as uniformly as possible. The cross-sections were mounted on sample stubs with the exposed surface facing upward and then coated with platinum using an ion sputter coater (Cressington 108 Auto). Morphology and particle size of starch granules were examined using an environmental scanning electron microscope (FEI, Model Q45). Multiple observation areas were randomly selected and imaged.

### 2.11 Identification of pollen viability using the iodine-potassium iodide staining method

Mature anthers were taken from the upper, middle, and lower parts of rice panicles at the onset of flowering. These anthers were left at room temperature for 0h, 3h, and 6h, respectively, then transferred onto microscope slides. Each anther was gently crushed with forceps, and 1-2 drops of 1% K-KI solution were added using a Barton’s dropper to fully release the pollen grains. A coverslip was placed over the sample and gently pressed with forceps, followed by a 2-3 min staining. A 10x microscope objective was used to observe randomly selected fields to examine anther morphology and record the proportion of mature anthers. The results were used to assess differences in pollen viability between transgenic lines and the recipient variety.

### 2.12 Field biodiversity evaluation

Field insect diversity survey: When the T2 generation rice cultivated outdoors reached the milky stage, three consecutive rain-free days were selected for sampling. Sticky traps were placed around each rice strain plot, with three random sampling points per strain and two sticky traps at each point. After three days, the sticky traps were collected, and the trapped insects were identified and counted to provide a preliminary assessment of pest presence during the reproductive growth stage.

Field plant diversity survey: For the rice cultivated outdoors, plant diversity was surveyed at the tillering stage (immediately after transplanting) and at maturity. At each stage, 3-5 plots per line were randomly selected. All plants within a 0.25 m² area (50 cm × 50 cm) surrounding each plot were collected, and their species diversity and biomass were recorded to preliminarily assess changes in plant diversity before and after planting of the transgenic materials.

### 2.13 Statistical analysis

Data are presented as the mean ± standard error of the mean (SEM). P values were calculated using one-way ANOVA. All graphs were generated using GraphPad Prism version 8.0.

## 3 Results

### 3.1 Stable expression of E2 antigen in different generations of rice

**Figure 1:**
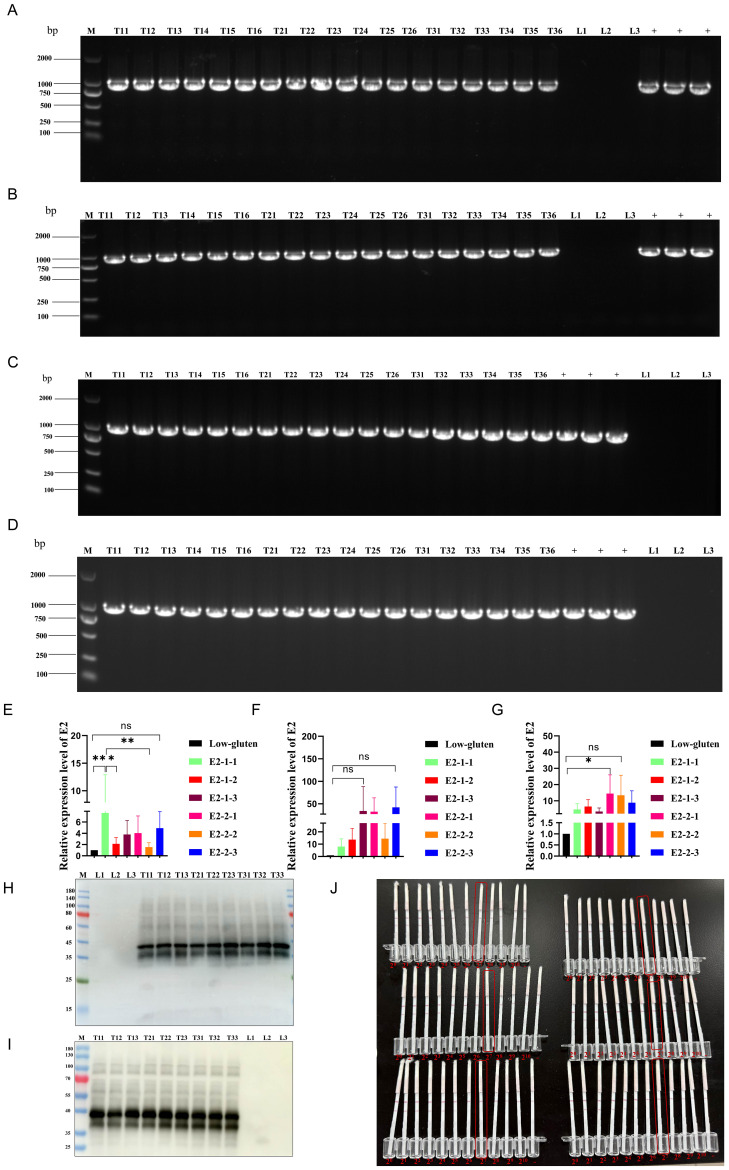
Stable presence of E2 gene in transgenic rice across generations as detected by PCR. **(A, C)** PCR (amplicon size: 985 bp) was used to detect E2 genes in rice leaves at the flowering stage and in rice spikes in different generations from E2-1, T11-T16 denote six randomly selected rice strains from T1 generation, T21-T26 denote six randomly selected rice strains from T2 generation, T31-T36 denote six randomly selected rice strains from T3 generation, L indicates Low-gluten, + indicates positive plasmid (The labelling in the figures is based on this method); (**B, D)** PCR (amplicon size: 985 bp) was used to detect E2 genes in rice leaves at the flowering stage and in rice spikes in different generations from E2-2. **(E–G)** correspond to the T1, T2 and T3 generations, respectively. E2-1 and E2-2 refer to the two rice strains screened which both successfully introduced the E2 gene; E2-1-1, E2-1-2 and E2-1-3 refer to three biological replicates randomly selected by E2-1; and similarly, E2-2-1, E2-2-2 and E2-2-3 refer to three randomly selected biological replicates of E2-2. (Three randomly selected biological replicates for each generation). **(H)** Expression of E2 gene in rice seeds of different generations of E2-1 and **(I)** E2-2 by Western blot; T11-T13 denote three randomly selected rice strains from T1 generation, T21-T23 denote three randomly selected rice strains from T2 generation, T31-T33 denote three randomly selected rice strains from T3 generation, L1-L3 denote three randomly selected Low-gluten **(J)** Test strips were used to detect E2 antigen titres in rice seeds of different generations (mass: volume = 1:5).

To detect the stable expression of the E2 protein in T1-T3 generations, field-grown E2-1 and E2-2 lines were selected. Rice leaves and spikelets were randomly collected at the flowering stage. PCR analysis confirmed the presence of the E2 gene in both leaves (**Figures 1A, C**) and spikelets (**Figures 1B, D**). qPCR analysis detected the transcript level of E2 in leaves, showing low but detectable expression (**Figures 1E–G**). Western Blot analysis revealed that the E2 protein was stably expressed in rice seeds across different generations (**Figures 1H, I**). Immunochromatographic test strips showed an antigenic titre of 2^7 in the different generations (**Figures 1J**). These results indicated that E2 protein was stably expressed across different generations of transgenic rice at the DNA, mRNA, and protein levels.

### 3.2 Combined agronomic traits of T1-T3 generation transformant lines and low-gluten

**Figure 2:**
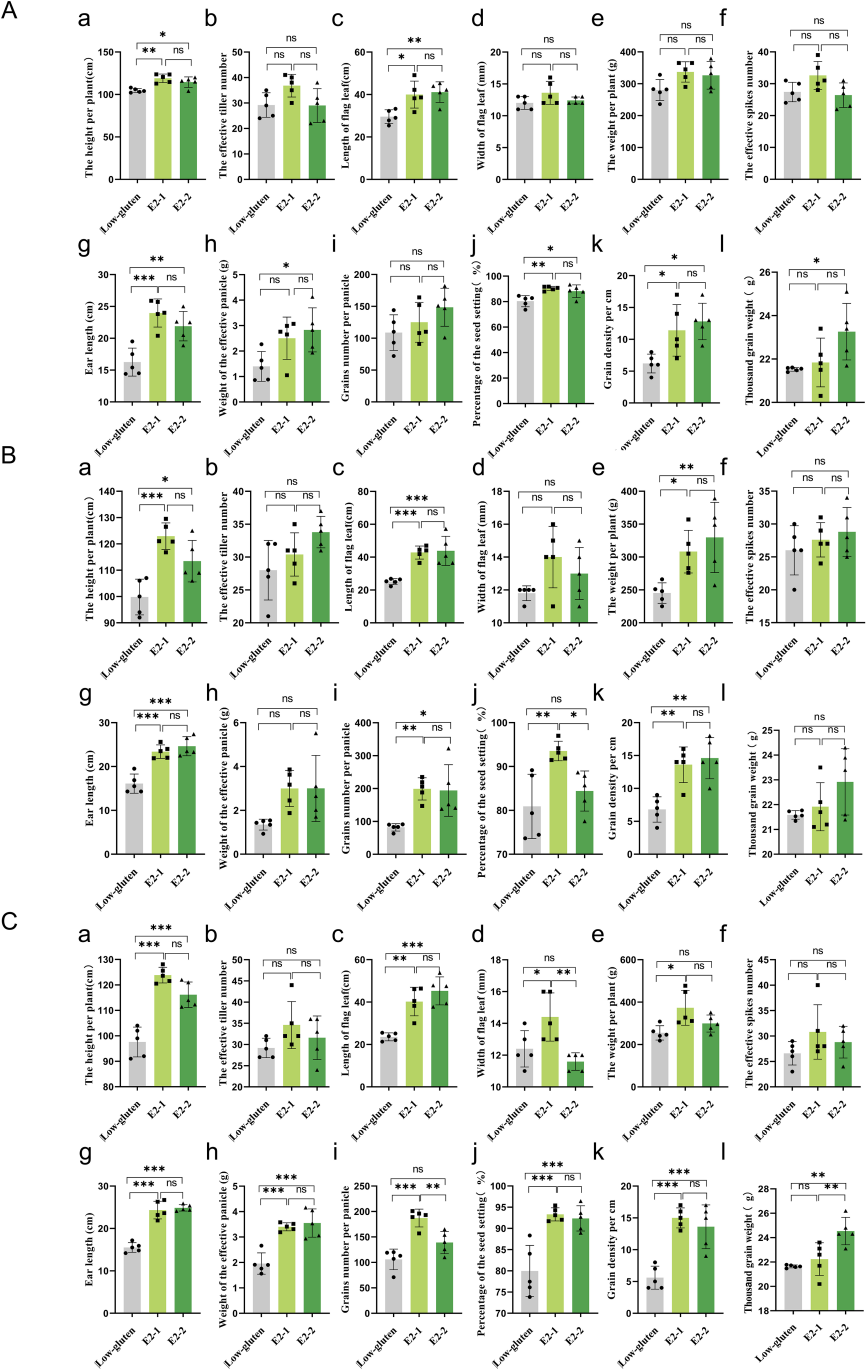
Combined agronomic traits of T1-T3 (E2-1 and E2-2 of the T1-T3 generations correspond to **(A–C)** generation transformant strains and Low-gluten. (a-c) Corresponded to 12 comprehensive agronomic traits recorded in T1-T3 generations, including: (a) The height per plant; (b) The effective tiller number; (c) length of flag leaf; (d) width of flag leaf; (e) The mass per plant; (f) The effective spikes number; (g) Ear length; (h) mass of the effective paicle; (i) grains number of per panicle; (j) percentage of the seed setting; (k) grain density per cm; and (l) Thousands grain mass; The data were statistically significantly different from those of the above data by One-way ANOVA, where * is *p*<0.05, **p<0.01, ***p<0.001, ****p<0.0001. Both E2-1 and E2-2 were assessed with five randomly selected strains.

During rice development, yield-related traits can be categorised into growth morphology, panicle status, and grain quality. This study assessed agronomic traits related to these three categories in T1-T3 generations. In the T1 generation, the E2-1 line showed greater plant height, flag leaf length, panicle length, seed setting percentage, and grain density per cm than the low-gluten in plant height, flag leaf length, grain length, effective panicle weight, seed setting percentage, grain density per cm, and thousand-grain weight. Other traits did not differ significantly. No significant differences in agronomic traits were observed between the two transgenic lines (**Figures 2A**). In the T2 generation, both E2-1 and E2-2 lines showed significantly higher plant height, flag leaf length, single plant weight, panicle length, number of grains per panicle, and grain density per cm than the low-gluten. The remaining traits were not significantly different. Comparing the two lines, E2-1 had a higher seed setting percentage than E2-2, while no significant differences were observed for the other traits (**Figures 2B**). In the T3 generation, E2-1 and E2-2 again showed higher plant height, flag leaf length, panicle length, effective panicle weight, seed setteing percentage, and grain density per cm than the low-gluten. Other traits were not significantly different. Comparing the two lines, E2-1 had greater flag leaf width and more grains per panicle, while E2-2 had a higher thousand-grain weight. The remaining traits showed no significant differences (**Figures 2C**).

### 3.3 Grain phenotypes of T1-T3 generation transformant lines and Low-gluten

**Figure 3:**
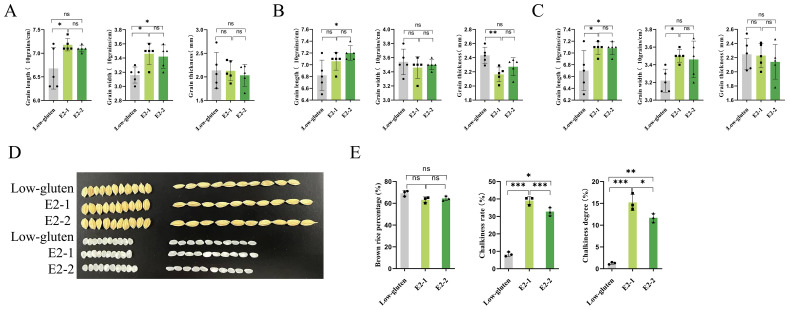
Grain phenotypes of T1-T3 generation transformant strains and Low-gluten. **(A–C)** Comparison of rice grain morphology data for each strain in the T1,T2 and T3 generation, from left to right, grain length, grain width and grain thickness (Both E2-1 and E2-2 were assessed with five randomly selected strains); **(D)** Rice grain phenotypes (brown rice on top, fine rice on bottom,n=10); **(E)** Comparison of rice grain quality among the strains, from left to right, in order of brown rice percentage, chalkiness and chalkiness (n=3). The data were statistically significantly different from those of the above data by One-way ANOVA, where * is *p*<0.05, **p<0.01, ***p<0.001, ****p<0.0001.

Grain quality was assessed in the T1-T3 generations by measuring grain length, grain width, grain thickness, brown rice rate, chalkiness rate, and chalkiness degree. In the T1 generation, both transgenic lines E2-1 and E2-2 showed a significant increase in grain width, while E2-1 also showed a significant increase in grain length compared to low-gluten. No significant differences in grain thickness were observed in either line (**Figures 3A**). In the T2 generation, E2-2 showed a significant increase in grain length, whereas E2-1 exhibited a significant decrease in grain thickness compared to low-gluten. No significant differences were observed between the two transgenic lines (**Figures 3B**). In the T3 generation, grain length was significantly increased in E2-1 and E2-2, and grain width was significantly increased in E2-1 compared to low-gluten. Grain thickness remained unchanged in E2-1. No significant differences were observed between the two lines. (**Figures 3C**). Grain morphology comparisons are further illustrated in **Figures 3D**, showing the grain length and width of brown rice (top) and milled rice (bottom) from the three lines. Additional assessments of brown rice rate, chalkiness rate, and chalkiness degree, which are also important characteristics of rice lines, are presented in **Figure 3E**. There were no significant differences in brown rice rate among the three lines; however, both E2-1 and E2-2 dislayed significantly higher chalkiness rate and chalkiness degree compared to low-gluten, especially in the E2-1 line.

### 3.4 Scanning electron microscopy analysis of grain chalkiness in low-gluten, E2-1, and E2-2 lines

**Figure 4:**
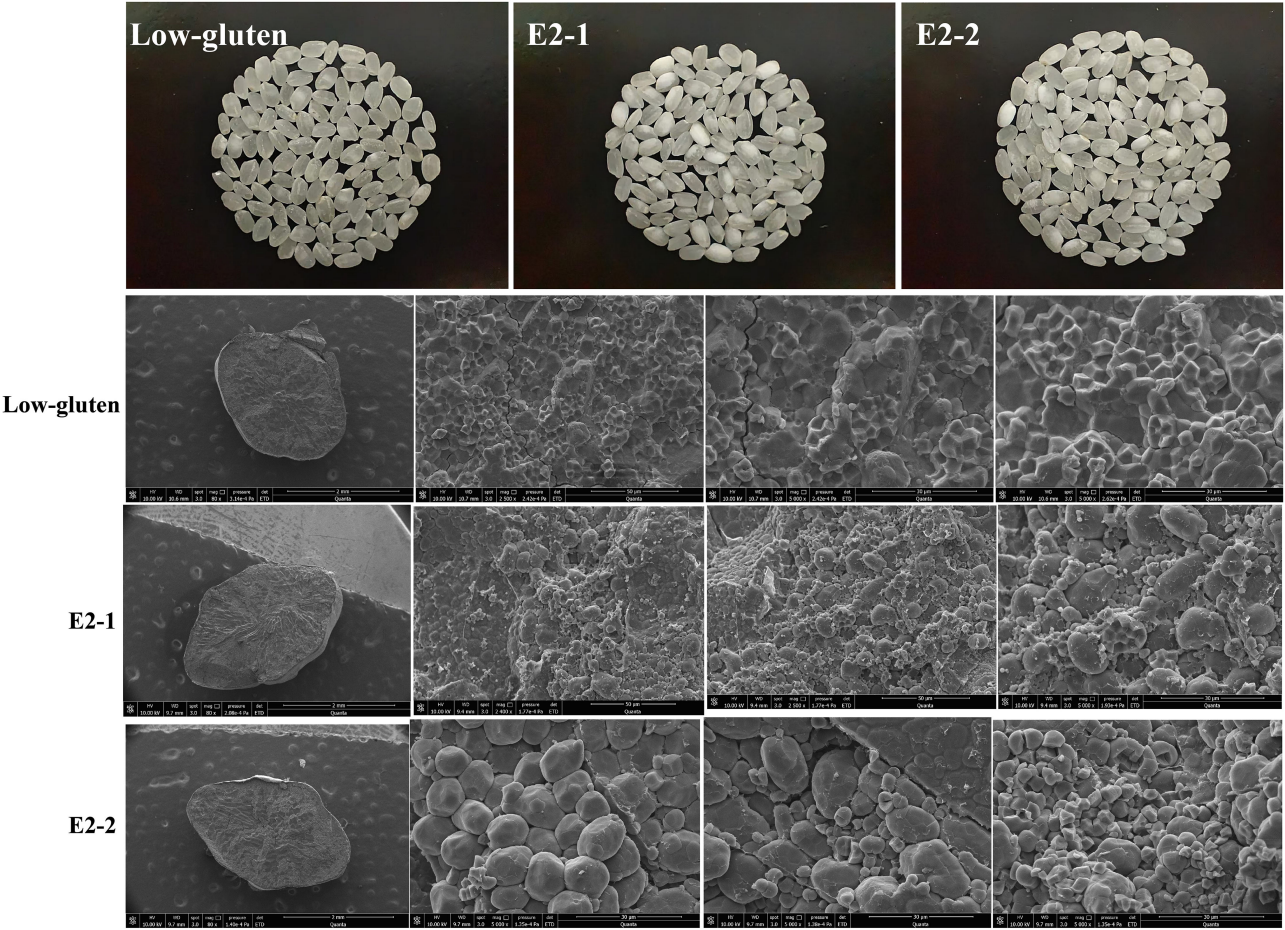
Scanning electron microscope detection of Low-gluten, E2-1, E2-2 respectively rice grain chalkiness (n=3).

To further confirm the chalkiness of the grains, the internal starch structure of rice grains was observed using scanning electron microscopy (**Figures 4**). Compared with the low-gluten, the E2-1 and E2-2 lines exhibited increased branched-chain starch, decreased straight-chain starch, and a looser starch arrangement. This structural difference contributed to higher brittleness in the grains. These findings are consistent with the observed increase in chalkiness rate and chalkiness degree in the E2 lines. Although chalkiness was more pronounced, the overall agronomic traits of the E2 lines remained favorable. This is a key advantage for efficient protein extraction via mechanical milling. Given these characteristics, the E2 line shows great potential for the industrial production of plant-derived vaccines through grain processing.

### 3.5 Germination and seedling percentage assessment of E2-1, E2-2 transgenic rice

**Figure 5:**
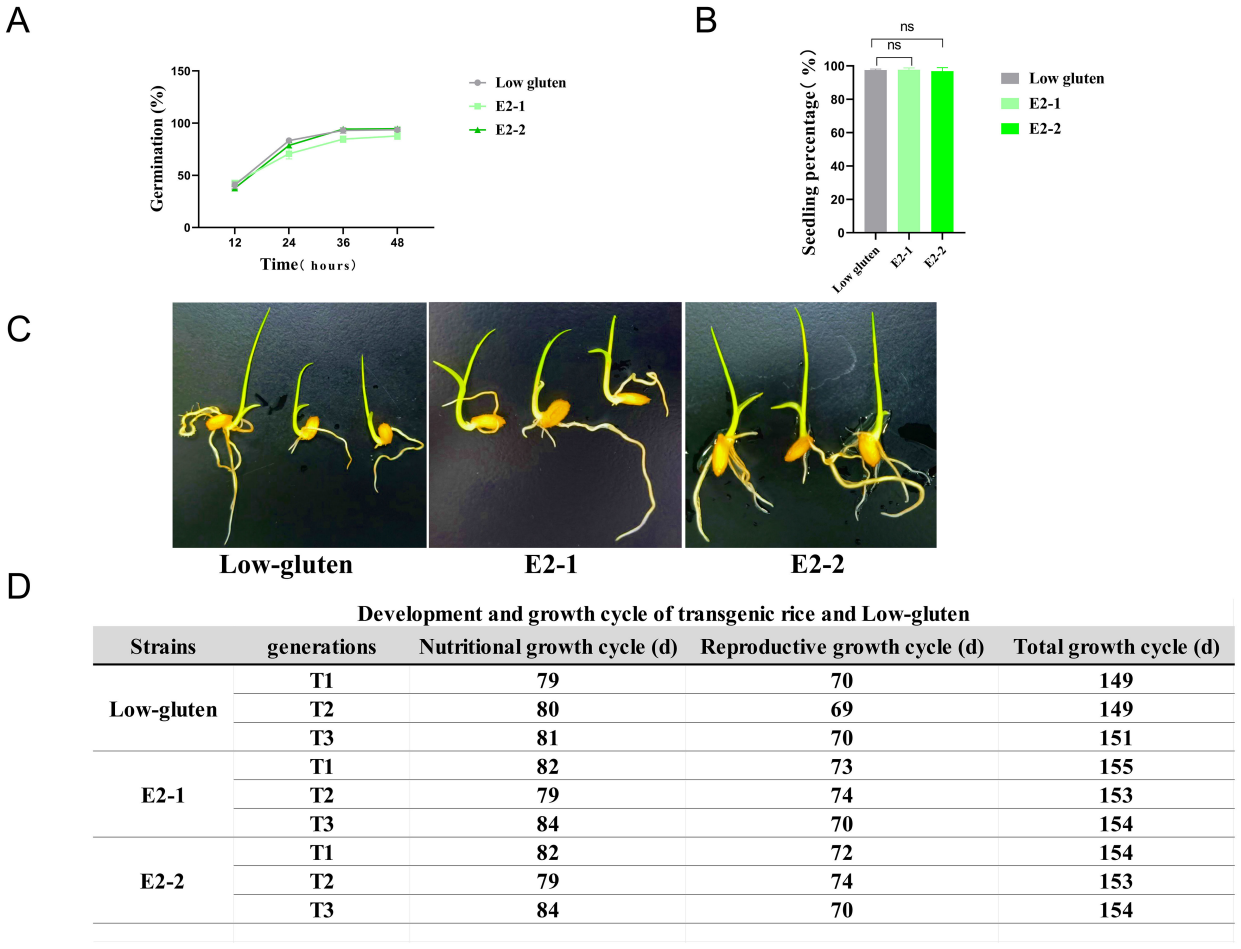
Germination and seedling percentage assessment of E2-1, E2-2 transgenic rice. **(A)** Visualisation of germination rates of E2-1, E2-2 and Low-gluten; **(B, C)** Germination and seedling percentage of transgenic rice (n=100); **(D)** Development and growth cycle of transgenic rice and Low-gluten.

To determine whether the transgenic rice lines E2-1 and E2-2 affect seed germination, seeds harvested at the same time were screened and germinated using the filter paper culture method. Germination data were observed and recorded at 12-hour intervals across four time points. The results showed no statistically significant differences in germination rates between E2-1, E2-2, and low-gluten (**Figures 5A, C**). Subsequently, seedlings were grown using the soil pot method, and emergence rates were recorded. The seedling emergence rate reached 90% for all lines (**Figures 5B**), with no significant differences between the transgenic lines and low-gluten. The total growth cycle was also similar, showing no significant changes. To investigate whether transgenic rice lines influence the duration of the growth cycle, E2-1 and E2-2, and low-gluten plants were cultivated outdoors for three consecutive generations. Their growth cycles were followed and recorded. The results showed that the total growth cycle of all lines remained stable, ranging from 145 to 155 days (**Figures 5D**). Both the vegetative and reproductive growth phases of E2-1 and E2-2 in the T1-T3 generations were comparable to those of the wild type. These results indicate that, under nutrient-sufficient conditions, the total growth cycles of transgenic rice lines E2-1 and E2-2 were comparable to those of low-gluten.

### 3.6 Comparison of pollen viability between transformant strains of T1-T3 generations and low-gluten

**Figure 6:**
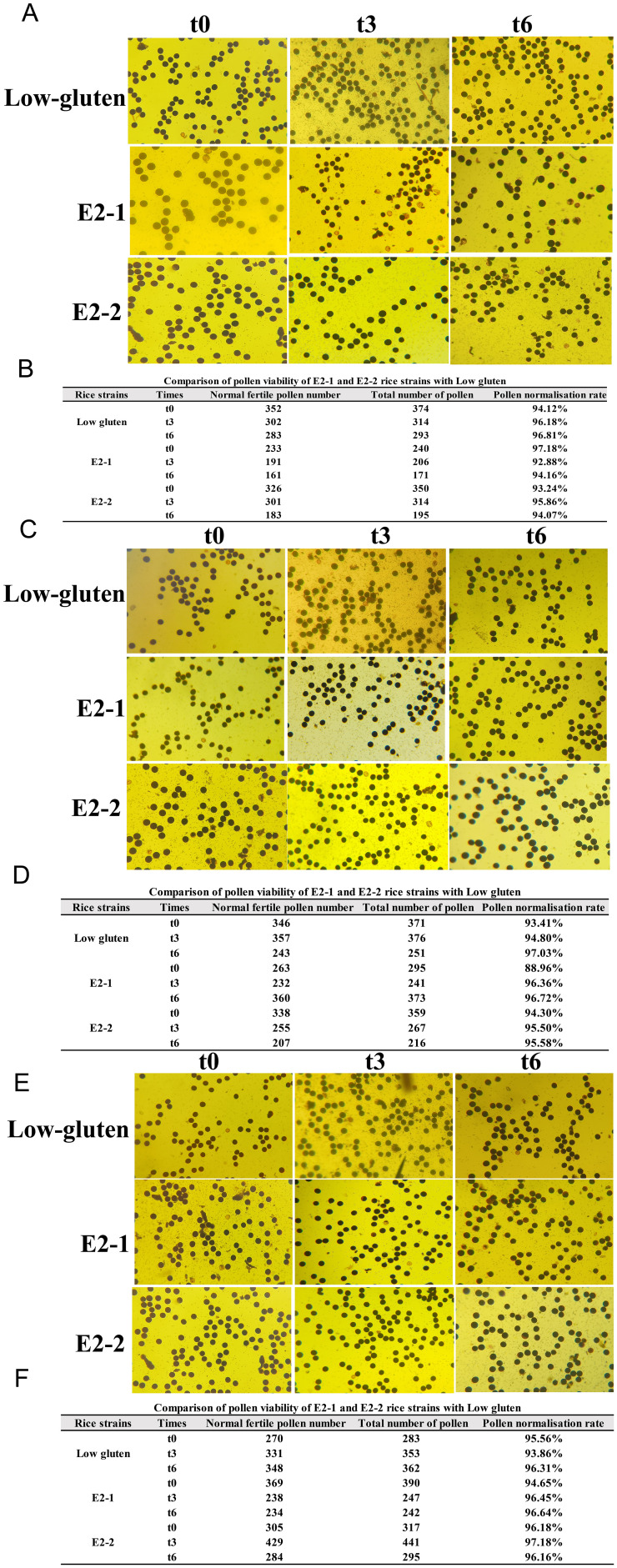
Comparison of pollen viability between transformant strains of T1-T3 generations and Low-gluten. **(A, B)** corresponds to the comparison and statistics of pollen viability of T1 generation transformant strain and Low-gluten, respectively; **(C, D)** corresponds to the comparison and statistics of pollen viability of T2 generation transformant strain and Low-gluten, respectively; **(E, F)** corresponds to the comparison and statistics of pollen viability of T3 generation transformant strain and Low-gluten, respectively.

Rice generally produces seeds through self-pollination, and pollen viability is a critical factor influencing reproductive ability and competitive survival in the field. To evaluate the reproductive competitiveness of the E2-1 and E2-2 transgenic lines compared to the low-gluten under field conditions, mature anthers collected at the initial flowering stage were stained with iodine-potassium iodide. Pollen viability was assessed at three time points (t0, t3, and t6). Across the T1 (**Figures 6A, B**), T2 (**Figures 6C, D**), and T3 (**Figures 6E, F**) generations, no significant temporal trends or differences were observed in the percentage of normal pollen between the transgenic lines and low-gluten. The proportion of fertile, viable pollen remained consistent among E2-1, E2-2, and low-gluten at each time point. The results indicated that the pollen longevity and viability of transgenic rice lines E2-1 and E2-2 was stable and comparable to those of low-gluten. Consequently, no significant difference in pollen viability was observed between the transgenic lines and low-gluten, and their competitive ability for survival in the field was consistent.

### 3.7 Safety evaluation of E2-1 and E2-2 transgenic rice lines

**Figure 7:**
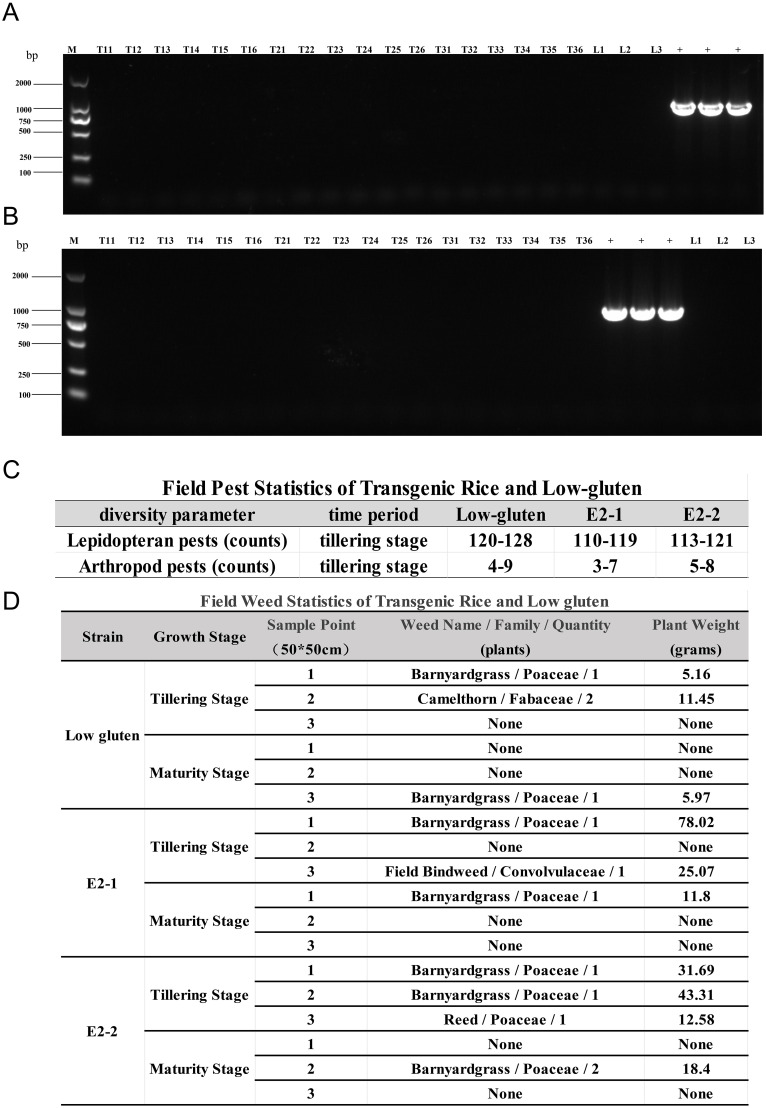
Safety evaluation of rice strains transgenic for E2-1 and E2-2 genes. **(A, B)** PCR result of the spread of exogenous gene to surrounding weeds from E2-1 and E2-2. T11-T16 denote six randomly selected rice strains from T1 generation, T21-26 denote six randomly selected rice strains from T2 generation, T31-36 denote six randomly selected rice strains from T3 generation, L indicates Low-gluten, + indicates positive plasmid (The labelling in the figures is based on this method); **(C, D)** Outdoor field biodiversity analysis of transgenic rice and Low-gluten.

Leaf samples from various weeds were collected from outdoor rice cultivation plots to assess potential gene transfer. PCR detection using E2-specific primers was performed to identify the presence of swine fever virus E2 gene in non-rice plants. As illustrated in **Figures 7A, B** no evidence of gene transfer was detected in any of the collected weed samples. Only the positive plasmid control showed a band containing the exogenous E2 gene, indicating that the gene was absent in all tested weed species. To further evaluate whether transgenic rice affects the population or abundance of surrounding organisms in the field, an insect diversity assessment was conducted. Mild-ripening rice plants with higher insect activity were selected, and armyworm traps (50 cm × 50 cm) were placed within both the wild type and transgenic rice blocks. After three days, the numbers of Lepidoptera were more prevalent than arthropod in all plots; however, no significant differences were observed in insect species composition or abundance between the low-gluten control and the E2-1 and E2-2 transgenic lines (**Figures 7C**). In the plant diversity survey, Random sampling of low-gluten and transgenic plots was conducted to record the species and weight of all other plants at transplanting and harvesting stages. The results revealed that, compared with the low-gluten control, the species and quality of weeds surrounding the transgenic strains E2-1 and E2-2 showed no significant differences (**Figures 7D**). These findings preliminarily suggest that the transgenic rice lines have minimal effect on the biodiversity of the surrounding environment. Importantly, no swine fever virus E2 gene transfer was detected in weeds from the cultivation plots, confirming effective containment of the transgene.

## 4 Discussion

Swine fever is an acute, febrile, and highly contagious infectious disease caused by CSFV infection. It is mainly characterized by rapid onset, sustained high fever, and degeneration of small blood vessel walls, causing widespread petechial haemorrhages, infarction, and tissue necrosis. Due to its high transmissibility and case fatality rate, the disease poses a severe threat to the pig industry. World Organization for Animal Health (OIE) classifies it as a notifiable disease, while China designates it as a Class I animal disease (Moennig, 2000; Giangaspero and Zang, 2023). Although the virulence of CSFV strains varies, all cause significant economic losses to the pig industry and pose a threat to pork production and food security globally. The disease remains endemic in parts of Asia, Central and South America, and several Eastern European countries, with occasional outbreaks in Western Europe. Currently, there are no effective antiviral treatments for swine fever; hence, vaccination and strict biosecurity remain the main strategies for prevention and control (Hua et al., 2014; Huang et al., 2014; Ji et al., 2014, Ji et al, 2015; Muñoz-González et al., 2015; Tong et al., 2022, Tong et al, 2024). Vaccines, as one of the major achievements of modern medicine, continue to play a central role in disease prevention. This study presents a comprehensive evaluation of a pre-commercial, rice-derived E2 protein vaccine for swine fever, focusing on genetic stability, environmental safety, and field adaptability. CSF, caused by CSFV, is a major threat to the global swine industry. Traditional vaccine production methods typically rely on mammalian or insect cell culture systems. However, these systems are expensive, susceptible to variations in antigen expression, and carry the risk of contamination with exogenous virus (Zhang et al., 2014; Dräger et al., 2015). In contrast, plant-based bioreactor systems, particularly the rice endosperm expression system, offer a promising alternative due to their low-cost production, high biosafety, and elimination of complex cold-chain storage and transport requirements.

Previous animal experiments showed that the swine fever E2 protein expressed in rice endosperm elicited a protective immune response in pigs. A low dose of 284 ng of the E2 vaccine induced neutralising antibody titres 32-fold higher than those generated by the live attenuated vaccine. In addition, the duration of E2-specific antibody-mediated immunity exceeded 180 days (Xu et al., 2023). These results confirmed the antigenic stability and strong immunogenicity of the rice-derived E2 protein. To further evaluate the feasibility of this plant-based vaccine platform, we conducted a comprehensive assessment of genetic stability, agronomic traits, and biosafety. Our results demonstrated stable E2 protein expression across the T1-T3 generations of transgenic rice. PCR analysis confirmed the presence of the E2 gene in both rice leaves and panicles. qPCR revealed low but detectable levels of E2 gene transcripts in the leaves. Western blot analysis consistently detected E2 protein expression in rice seeds from different generations, with antigen titres maintained at 2^7. These findings underscore the genetic stability of the E2 protein expression in the rice endosperm system, a crucial requirement for commercial development. Additionally, to assess the impact of transgenic modification on agronomic traits, we compared the E2-1 and E2-2 lines with the low-gluten control. Our results showed no statistically significant differences in seed germination-related traits between the transgenic lines and the control. Additionally, the transgenic lines exhibited comparable or even enhanced agronomic traits, including growth morphology, panicle status, and grain quality. Notably, the E2 lines showed increased grain length and width, which, although accompanied by higher chalkiness, are advantageous for efficient protein extraction through mechanical milling. The total growth cycles of the transgenic lines were stable and comparable to those of the wild type, indicating no adverse effects on rice growth and development. Biosafety assessment is critical for for the commercialization of transgenic crops. Field experiments were conducted evaluate the survival competitiveness and potential gene flow of the transgenic rice lines. Pollen viability analysis revealed no significant differences in pollen longevity and fertility between the transgenic lines and the low-gluten control, suggesting ecological equivalence in reproductive competitiveness. Crucially, PCR analysis of weed samples collected from the cultivation plots revealed no evidence of E2 gene transfer, confirming stringent containment of the transgene and the environmental safety of the transgenic rice lines. Moreover, insect and plant diversity experiments indicated minimal impact of the transgenic rice on the surrounding biodiversity. These findings provide further support for the environmental safety and biosafety of the rice-based platform for swine fever E2 protein production.

Traditional swine fever vaccine production platforms rely heavily on mammalian or insect cell culture systems, which are costly and susceptible to antigenic variation and exogenous viral contamination. In contrast, the rice endosperm expression system offers a low-cost, high-efficiency, and biosafe alternative for vaccine production. The stable expression of the E2 protein in transgenic rice, along with favorable agronomic traits and minimal biosafety risks, highlights the strong potential of this platform for the commercial production of swine fever vaccines.

Importantly, the rice endosperm expression system offers several key advantages over traditional vaccine production platforms, including low-cost production, high biosafety, and the elimination of complex cold-chain storage and transportation requirements (Giddings et al., 2000). These advantages make it particularly well-suited for use in resource-limited regions, where access to traditional vaccines is often hindered by cold-chain constraints. This study lays the foundation for the commercialisation of plant-derived swine fever vaccines based on the rice endosperm system. The successful expression and immunogenicity of the E2 protein demonstrate the antigenic stability and effectiveness of this platform. Furthermore, the comprehensive evaluation of genetic stability, agronomic traits, and biosafety confirms its feasibility and safety for large-scale vaccine production. The successful production of bioactive swine fever virus E2 protein via the rice endosperm expression system highlights its potential as a cost-effective and environmentally friendly vaccine solution for swine fever prevention and control. The stable expression of the E2 protein, along with favorable agronomic traits and verified biosafety, supports its scalability and commercia viability. Furthermore, this study establishes a technological model for developing vaccines against livestock and poultry diseases using crop-based bioreactors, promoting cross-disciplinary innovation between plant biology and veterinary science.

## 5 Conclusion

In summary, this study successfully produced bioactive swine fever virus E2 protein using the rice endosperm expression system and verified its advantages in antigenic stability, agronomic traits, and biosafety. This technology holds potential for further development and application in the formulation of other vaccines. As an efficient and cost-effective platform for protein production, the rice endosperm expression system not only shows promise in vaccine development but also provides important references for the production of other medicinal proteins.

## Data Availability

The original contributions presented in the study are included in the article/supplementary material. Further inquiries can be directed to the corresponding authors.
